# Composites Based on Nanoparticle and Pan Electrospun Nanofiber Membranes for Air Filtration and Bacterial Removal

**DOI:** 10.3390/nano9121740

**Published:** 2019-12-06

**Authors:** Ana Cláudia Canalli Bortolassi, Vádila Giovana Guerra, Mônica Lopes Aguiar, Laurence Soussan, David Cornu, Philippe Miele, Mikhael Bechelany

**Affiliations:** 1 Departamento de Engenharia Química, Universidade Federal de São Carlos–UFSCar, Rodovia Washington Luiz, km 235–SP 310, São Carlos 13565-905, Brazil; acanallibortola@deakin.edu.au (A.C.C.B.); vadila@ufscar.br (V.G.G.); mlaguiar@ufscar.br (M.L.A.); 2 Institut Européen des Membranes, IEM–UMR 5635, ENSCM, CNRS, Univ Montpellier, 34070 Montpellier, France; laurence.soussan@univ-montp2.fr (L.S.); david.cornu@enscm.fr (D.C.); philippe.miele@umontpellier.fr (P.M.)

**Keywords:** air filtration, nanoparticles, electrospinning

## Abstract

Often, solid matter is separated from particle-laden flow streams using electrospun filters due to their high specific surface area, good ability to capture aerial particulate matter, and low material costs. Moreover, electrospinning allows incorporating nanoparticles to improve the filter’s air filtration efficiency and bacterial removal. Therefore, a new, improved polyacrylonitrile (PAN) nanofibers membrane that could be used to remove air pollutants and also with antibacterial activity was developed. We engineered three different filters that are characterized by the different particles embedded in the PAN nanofibers: titanium dioxide (TiO_2_), zinc oxide (ZnO), and silver (Ag). Then, their filtration performance was assessed by quantifying the filtration of sodium chloride (NaCl) aerosol particles of 9 to 300 nm in diameter using a scanning mobility particle sizer. The TiO_2__F filter displayed the smallest fiber diameter and the highest filtration efficiency (≈100%). Conversely, the Ag_F filter showed the highest quality factor (≈0.06 Pa^−1^) because of the lower air pressure drop. The resulting Ag_F nanofibers displayed a very good antibacterial activity using an *Escherichia coli* suspension (10^8^ CFU/mL). Moreover, the quality factor of these membranes was higher than that of the commercially available nanofiber membrane for air filtration.

## 1. Introduction

Fossil fuels and industries release many pollutants in the atmosphere [1]. Particles smaller than 2.5 µm (PM_2.5_) are particularly dangerous for humans because their small size facilitates their diffusion in bronchi and lungs [2]. The particulate matter’s size is determined by how such particles are produced. For example, the size of combustion particles is normally about 10–50 nm; however, they can combine with other particles and generate larger particulates. All these agglomerated particles can be released in the air when broken down. Complex mixtures of particles, most of them usually with a diameter smaller than 1000 µm, are the contaminants that are eliminated by air filtration. The diameter of particles in chemical and biological aerosols varies between 1 and 10 µm (particles smaller than 2.5 µm are particularly dangerous for human health).

Therefore, industries that need to produce material with the lowest possible amount of impurities are very interested in filters that can trap particulate and biological contaminants. Indeed, the size of the industrial air-filtration market should exceed USD 6.5 billion by 2024 [3]. However, the existing high-efficiency air filters cannot block particles smaller than 3 µm in diameter or small pathogenic agents, such as viruses that are smaller than 1 µm [4]. Particles of diameter smaller than a unit density sphere take more time to settle in air compared with bigger particles [5]. Therefore, advanced filtration technologies are needed to capture such nanometric particles. Moreover, heating, ventilating, and air conditioning air filters that are designed to purify air in wet, dark, and ambient temperature conditions are more likely to be colonized by bacteria, molds, and fungi. This leads to bad odor and poor air quality [6]. Therefore, new filters need to be developed or existing filters need to be improved due to the increased resistance of microorganisms and also the limits of their antimicrobial activity [4].

Currently, membrane filtration is thought to be the most effective physical approach against air pollutants [7]. Nanofiber membranes can trap most contaminants and can qualify as high-efficiency particulate air (HEPA) filters. Moreover, they are characterized by low air pressure drop and basis weight as well as compact structure. The filter efficiency in separating particles from the air stream is influenced by the particle composition, shape, filtration velocity, and type of impaction surface [8]. Membrane filters rely on physical interactions to efficiently separate and collect particles. Data from many different studies led to the general conclusion that filtration efficiency increases with high basis weight and decreases with higher superficial flow velocity [9,10,11].

Therefore, novel fibrous filters with lower energy consumption for air purification require the development of resistant fibers with high filtration efficiency and low pressure drop [12]. Electrospinning is a highly popular method to produce many different fiber morphologies including very fine diameters, various porosities and pore sizes (from nanometers to micrometers), and great mechanical strength, thanks to inter-fiber connections [13,14,15,16,17]. A surface modification can enhance electrospun nanofibers when nanoparticles are incorporated in membranes.

Some studies have already investigated the addition of active nanoscale materials, such as SiO_2_, Al_2_O_3_, and CuO, in electrospun fibers to improve their air pollutant filtration capacity and also increase their mechanical, thermal, and chemical resistance [18,19,20]. Several groups have focused on titanium dioxide (TiO_2_) and zinc oxide (ZnO) due to their potential as antibacterial agents, mainly linked to their small particle size, high surface area, photocatalytic bactericidal activity, self-cleaning properties, and low cost [21,22]. After their adsorption to the bacterial cell surface, Zn and Ti ions cross the cell membrane and cause cell disruption, DNA damage, protein activity inhibition, and ultimately cell death [23,24,25]. Silver (Ag) nanoparticles and salts also display particularly attractive antimicrobial effects. Indeed, they are non-toxic to human cells but are highly effective against bacteria, fungi, and viruses [26]. In addition, Ag nanoparticles’ bactericidal properties against a wide range of microorganisms are mediated through the production of reactive oxygen species [27]. However, the aggregation and dissolution of Ag, TiO_2_, and ZnO nanoparticles are still a challenge for practical application [28].

Polyacrylonitrile (PAN) has been widely used for the production of membranes because it displays a very good mechanical strength and chemical stability [29]. Several studies have investigated nanofibers made of polymers and bactericidal particles to be used as air filters [30,31,32]. Moreover, Ag, TiO_2_, and ZnO antimicrobial activity has been widely studied [33,34,35,36]. However, to our knowledge, no study has concomitantly analyzed their bactericidal effect and ability to trap NaCl particles smaller than 300 nm. Sim et al. [37] showed a high antimicrobial activity (>99%), filtration efficiency (~92.5% against a 300 nm KCl aerosol), and small pressure drop (~0.8 mmAq at 13 cm/s) of an antimicrobial-nanoparticle-coated electrostatic air filter in an indoor environment. Lv et al. [38] analyzed the performance of nanofiber membranes composed of poly(vinyl alcohol) (PVA) and konjac glucomannan (KGM), and loaded with ZnO nanoparticles for air filtration and water treatment. These nanofiber membrane filters showed an efficient air-filtration performance (>99.9% for ultrafine particles, 300 nm), excellent photocatalytic activity, and antibacterial activity against Gram-negative (*Escherichia coli*) and Gram-positive bacteria (*Bacillus subtilis*). Recently, we described novel electrospun Ag/PAN fibers that could be used as air filters for nanoparticle removal, and that showed antibacterial activity [39].

Here, we used electrospinning to fabricate novel PAN membranes loaded with active ZnO, TiO_2_, or Ag nanoparticles to increase their air-filtration efficiency, lower the pressure drop, and improve their quality factor, and exhibit bactericidal activity. Moreover, we analyzed the fiber distribution to understand its influence on the filtration of sub-300-nm particles.

## 2. Materials and Methods

### 2.1. Materials

PAN (M_w_~150,000 g/gmol; CAS Number 25014-41-9), N,N-dimethylformamide (DMF; 99.8%; CAS number 68-12-2), TiO_2_ (M_w_~79,87 g/gmol; CAS number 13463-67-7), ZnO (M_w_~81,39 g/gmol; CAS number 1314-13-2), and silver nitrate (AgNO_3_; M_w_~169.87 g/gmol; CAS number 7761-88-8) were bought from Sigma Aldrich (St. Louis, MO, USA). The substrate made of polyethylene terephthalate (PET) fibers and used to collect nanofibers was from Freudenberg (Weinheim, Baden-Württemberg, GE). NaCl (99%; CAS number 7647-14-5) was from Sigma Aldrich (St. Louis, MO, USA) and was used to produce nanoparticles to determine the membrane’s filter-removal efficiency.

### 2.2. Methods

#### 2.2.1. Preparation of TiO_2_/PAN/DMF, ZnO/PAN/DMF, and Ag/PAN/DMF Solutions

Nanofibers were produced by dissolving 0.95 g of PAN polymer (9.1 wt%) in 10 mL of DMF. After 2 h of agitation, 0.95 g of TiO_2_, ZnO, or AgNO_3_ (the same amount as for PAN) were added in the PAN solution. The average size of the obtained TiO_2_, ZnO, and Ag nanoparticles was 21, 50, and 5 nm, respectively, according to the supplier. Solutions were continuously stirred in the dark and at room temperature for 48 h to form a homogenous solution. This period of time was necessary to allow AgNO_3_ reduction to Ag nanoparticles using DMF as solvent at room temperature and without any reducing agent [40,41,42]. Ag nanoparticles changed color due to the surface plasmon resonance (SPR) phenomenon that occurs when light is reflected off a thin metal film or nanoparticles [36]. Conversely, the ZnO/PAN/DMF and TiO_2_/PAN/DMF solutions did not change color after stirring for 48 h. Viscosity was measured with a Brookfield viscometer spindle 29 (TC-650, AMETEK Brookfield, Middleborough, MA, USA) and conductivity with an electrical conductivity meter (TEC-4MP, Tecnal, Piracicaba, San Paulo, Brazil).

#### 2.2.2. Fabrication of TiO_2_/ZnO/Ag-PAN Nanofibers by Electrospinning

The different nanoparticle/PAN/DMF solutions were loaded in 12 mL syringes with 0.7 mm diameter needles and were fed (flow rate = 0.2 mL/h) into the home-made electrospinning system using a syringe pump (KDS 100, KD Scientific, Holliston, MA, USA) [43,44,45] powered (25 kV) by a High Voltage Power Supply (T1CP 300 304n-iSeg, Radeberg, Germany). Nanofibers were deposited on PET films (i.e., the substrate) wrapped around the rotating machine. The syringe tip-collector distance was set at 15 cm. The prepared filter media were named Ag_F, TiO_2__F, ZnO_F, and PAN_F when AgNO_3_, TiO_2_, ZnO, and no nanoparticles were added to the PAN solution, respectively. During electrospinning, the high applied voltage was obtained by connecting the positive and ground terminals to the nozzle and collector, respectively. When the jet exceeded a critical voltage, a stable jet of liquid was ejected from the syringe tip. After solvent evaporation, nanofibers were produced and deposited on the collector surface where the non-woven substrate was placed. This process was carried out at approximately 20 °C under atmospheric air and the substrate was on a grounded metal roller (10 cm in diameter) that rotated at 300 rpm for 1 h for each experiment.

#### 2.2.3. Structural and Morphological Analysis of Nanofiber Filters

Energy-dispersive X-ray (EDX) spectroscopy and elemental mapping were performed using a Zeiss EVO HD15 microscope (Oberkochen, Germany) coupled to an Oxford X-MaxN EDX detector (Oxford Instruments, Abingdon, UK) to measure the atomic percentage (Figure S1). The nanofiber diameter distribution was analyzed by scanning electron microscopy (SEM) with a Hitachi S4800 microscope (Tokyo, Japan), and thickness was measured with a caliper (Starrett, Athol, MA, USA). The fiber diameter was measured from SEM images using Image J1.29X as described by Bortolassi et al. [46]. The fiber size distribution was determined by measuring 100 fibers of each filter medium.

An attractive air filter should display low pressure drop and high permeability. Permeability was assessed by varying the air velocity from 0.3 to 3 cm/s, and pressure drop (Δ*P*) was quantified with a digital manometer (VelociCalc Model 3A-181WP09, TSI, Shoreview, MN, USA) linked to the filtration apparatus, as previously described [46].

As the filtration velocity used in this study was low, the permeability constant ($k_{1}$) was calculated using Darcy’s law that analyzes the fluid flow of the filter media relative to the pressure drop (ΔP), thickness (*L*), air viscosity (μ), and superficial velocity ($v_{s}$):

(1) $$\frac{\Delta P}{L}=\frac{\mu }{k_{1}}v_{s}$$

The pore-size distributions of the webs was assessed with a capillary flow porometer (3 gzh Quantachrome, Anton Paar, Graz, Austria), as previously described [47]. The pore distribution, mean pore size, and bubble point were measured at pressure values ranging from 0.3 to 0.8 bar and using isopropanol as wetting liquid. The pore size–pressure relationship is described by Equation (2):

(2) $$P=\frac{4\gamma_{l/g}Cos\theta }{d}$$

where, *P* is the applied pressure, $\gamma_{l/g}$ is the wetting liquid surface tension, θ is the wetting angle, and *d* is the pore diameter.

#### 2.2.4. Testing the Nanofiber Filter’s Filtration Performance

Nanoparticles of different sizes (from 9 to 300 nm) were generated using 0.1 g/L of NaCl solution and an atomizer aerosol generator to obtain the same, standard particle size distribution curve for all tested filters.

The experimental unit included an air compressor (Shultz, Acworth, GA, USA), air purification filters (Model A917A-8104N-000 and 0A0-000), an atomizer aerosol generator (Model 3079, TSI, Shoreview, MN, USA), a diffusion dryer (Norgren IMI, Birmingham, UK), a Kriptônio and Americium neutralizing source (Model 3054, TSI, Shoreview, MN, USA), a filter apparatus, a flowmeter tube size 3 (Gilmont, Vernon Hills, IL, USA), and a scanning mobility particle sizer that comprises an electrostatic classifier (Model 3080, TSI, Shoreview, MN, USA), a differential mobility analyzer, and an ultrafine particle counter (Model 3776, TSI, Shoreview, MN, USA), as described by Bortolassi et al. [46].

The nanoparticle concentration upstream (*C_up_*) and downstream (*C_d_*) of the filter medium was calculated using the differential mobility analyzer and particle counter and the following equation:

(3) $$n_{t}=\frac{C_{up}-C_{d}}{C_{up}}$$

Filtration efficiency was tested at constant superficial velocity (0.05 m/s), flow rate (1.50 L/min), and filtration area (5.3 cm^2^). Experiments were repeated three times and data were presented as the mean ± standard deviation. The experimental and theoretical collection efficiency of the filter media were compared with Equation (4) [48]:

(4) $$n_{t}=n_{d}+n_{i}+n_{id}+n_{g}+n_{e}$$

where $n_{t}$ is the total collection efficiency, $n_{d}$ is the diffusion, $n_{i}$ the inertial, $n_{id}$ the interception, $n_{g}$ the gravitational, and $n_{e}$ the electrophoretic mechanism. For our test, diffusion, inertial, and interception are the most important mechanisms and are influenced by different parameters, particularly air velocity, fiber and particle diameter, and porosity.

Quality factor (*Q_F_*) also is used to describe the effectiveness of filter media. It was assessed by relating the pressure drop to the removal efficiency of 100-nm particles, as described by Equation (5) [48]:

(5) $$Q_{F}=\frac{-\ln\left(1-n\right)}{\Delta P}$$

where Δ*P* is the pressure drop across the filter and *n* is the removal efficiency.

The minimum efficiency at the most penetrating particle size typically ranges from 0.1 µm and 0.5 mm, and is influenced to various degrees by different mechanical mechanisms (e.g., interception, diffusion, and inertial impaction) involved in particle filtration [49]. Moreover, it is generally acknowledged that particles from an aerosol will be removed more efficiently by a thick filter, but with higher pressure drops. Conversely, removal by a thin, open-porous filter is usually less efficient, but this filter type is more permeable. Therefore, further increasing the filter efficiency could compensate for the higher pressure drop. Hence, high *Q_F_* values are the result of a balance between efficiency and pressure drop.

#### 2.2.5. Bactericidal Activity

Antibacterial tests were performed using non-pathogenic Gram-negative *E. coli* bacteria (K12 DSM 423, from DSMZ, Braunschweig, Germany). Lysogeny broth (LB) Miller was used for *E. coli* culture, counting, and direct-contact agar tests. For each experiment, a new bacterial suspension was prepared from frozen *E. coli* aliquots kept at −20 °C. After rehydration in LB medium at 30 °C on a rotating shaker (160 rpm) for 3 h, aliquots were inoculated in fresh LB medium (5% v/v) and incubated at 30 °C under constant stirring (160 rpm) overnight to reach the stationary growth phase. Then, bacteria were centrifuged (4000 rpm and 12 °C for 10 min) and supernatants were discarded to remove nutrients. Bacterial pellets were suspended in spring water (Cristaline Sainte Cécile, Sainte-Cécile, France: [Ca^2+^] = 39 mg/L, [Mg^2+^] = 25 mg/L, [Na^+^] = 19 mg/L, [K^+^] = 1.5 mg/L, [F^−^] < 0.3 mg/L, [HCO_3_^−^] = 290 mg/L, [SO_4_^2−^] = 5 mg/L, [Cl^−^] = 4 mg/L, [NO_3_^−^] < 2 mg/L) to block bacterial growth. The bacterial concentration in the suspension was measured by absorbance measurements at 600 nm and adjusted according to a calibration curve previously prepared in the laboratory. The suspension was diluted with spring water to 10^8^ and 10^3^ CFU/mL. To test the antibacterial activity of ZnO/TiO_2_/Ag-PAN materials, direct-contact agar tests were done using nutritive LB agar plates. Briefly, 40 µL of bacterial suspension was deposited on sterile 2.25 cm^2^ (Ag_F, TiO_2__F, ZnO_F, and PAN_F) samples that were previously sterilized by UV exposure for 30 min. The inoculated side of the samples was placed on nutritive LB agar at room temperature for 6 h and then removed. Then, plates were incubated at 30 °C overnight to allow the growth of bacterial colonies (each bacterium should produce one colony). The bacterial log removal could also be calculated for the lowest bacterial concentration tested (i.e., 10^3^ CFU/mL). In the positive controls, the bacterial suspension was placed directly on LB agar plates without pre-incubation with any sample. Each test was performed in triplicate.

## 3. Results and Discussion

Electrospinning was used to fabricate PAN nanofiber filters (PAN_F) that included different bactericidal nanoparticles (Ag, TiO_2_, or ZnO). After nanoparticle addition (0.95 g of AgNO_3_, TiO_2,_ or ZnO) in the PAN solution (9.1 wt%), viscosity and conductivity were analyzed. The obtained filter media were named Ag_F, TiO_2__F, and ZnO_F, respectively. Then, the different features of PAN nanofibers and Ag/TiO_2_/ZnO-PAN nanofibers were analyzed (fiber distribution, thickness, porosity, permeability, and pressure drop) as well as their filtration performance, *Q_F_*, and bactericidal activity.

### 3.1. Solution Characterization

Quantification of conductivity and viscosity (Table 1) showed that after adding AgNO_3_ to the PAN solution, Ag nanoparticles were formed (confirmed by Bortolassi et al. [39]), and conductivity changed. Moreover, viscosity increase was much higher in the Ag_F solution than in the TiO_2__F and ZnO_F solutions. A very high viscosity could affect nanofiber deposition, as a result of the hard ejection of jets from the solution, as proposed by Li & Wang [50]. Therefore, the higher Ag_F conductivity and viscosity led to the deposition of thinner and lighter fiber layers compared with the other solutions. Moreover, high viscosity could hinder the formation of electrospun nanofibers.

### 3.2. Structural and Morphological Properties

**Figure 1:**
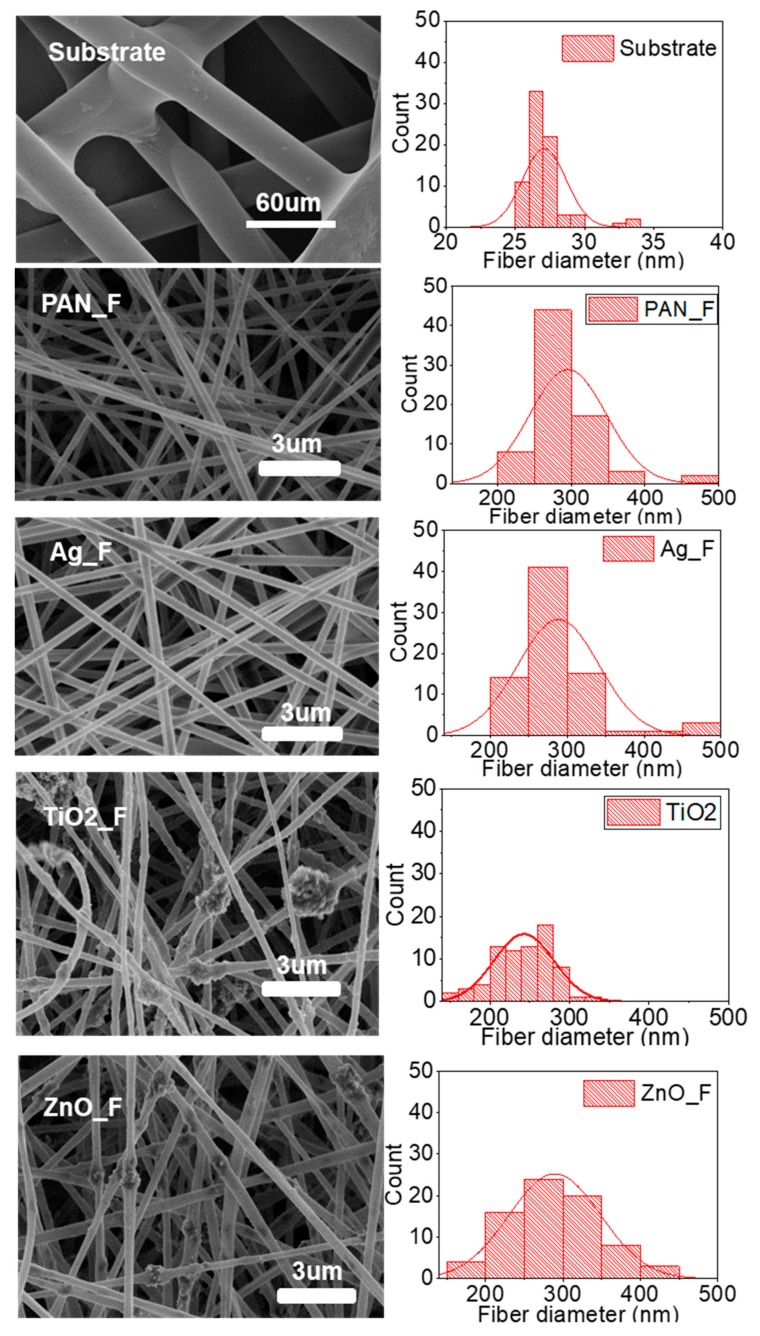
SEM images and fiber diameter distribution for the polyethylene terephthalate (PET) substrate and electrospun polyacrylonitrile nanofiber filter (PAN_F), Ag_F, TiO_2__F, and ZnO_F samples. The red lines show the approximate distribution based on a Gaussian distribution.

SEM imaging and size distribution analysis of Ag/TiO_2_/ZnO-PAN nanofibers after electrospinning (Figure 1) showed that PAN_F, Ag_F, and ZnO_F filters were composed of nanofibers with a mean diameter of 290 nm. TiO_2__F filters had the smallest fiber diameter (242 nm). The substrate used to support the nanofiber deposition was composed of PET microfibers with a mean fiber diameter of approximately 27 µm.

All filters fabricated in this study were composed of heterogeneous nanofibers, but only TiO_2_ filters included large nanoparticle agglomerations in the fibers. This could be explained by the large specific surface area and the interaction between TiO_2_ nanoparticles and PAN fibers. Indeed, TiO_2_ nanoparticle aggregation in the middle of fibers could have been favored by electrostatic repulsion of positively charged TiO_2_ by the positive charges on the fiber surface [51]. Wang et al. [52] also described the agglomeration of TiO_2_ nanoparticles upon production of PLA (7 wt%) fibers with lower concentration of TiO_2_ nanoparticles (0.5, 1, 1.5, 1.75, and 2 wt%) due to the lower nanopore volume. Lv et al. [33] dissolved 1 g of KGM and ZnO nanoparticles (0, 0.5, 1.0, 1.5, and 2.0 wt%) in PVA solution (10%) using ultrasonic stirring, and did not observe any agglomeration. Moreover, it has been reported that in PAN/TiO_2_ fibers, TiO_2_ nanospheres tend to agglomerate more readily due to their smaller sizes compared with ZnO nanoparticles [51]. Conversely, no cluster was observed upon Ag_F production, probably because Ag nanoparticles can easily spread along nanofibers due to their small size (5 nm).

As the morphology of electrospun nanofibers can be influenced by many factors, particularly the solution concentration, applied voltage, solution velocity, distance between syringe tip and collector distance, and solution properties (polarity, surface tension, electric conductivity), the same electrospinning conditions were used for all samples in our study. Agglomeration is also influenced by the electrospinning parameters, but this issue was not investigated in this study.

Measurement of the filter’s thickness (Table 2) did not highlight any significant difference after the deposition of the electrospun nanofibers on the substrate (S), because the nanofiber layer was very thin. Sample weighing after electrospinning indicated that the basis weight increase was higher in the TiO_2__F and ZnO_F than Ag_F samples, probably due to nanoparticle agglomeration. Moreover, the fiber diameter decreased when nanoparticles were added to the PAN solution, as observed by Abdo et al. [53]. The reduced diameter decrease [54] and lower basis weight [50] of the electrospun Ag_F samples can be explained by the higher viscosity and conductivity of the Ag/PAN/DMF solution.

The EDX analysis allowed calculating the atomic percentages of Ag/Ti/Zn in the fabricated fibers (Table S1). The obtained values are in agreement with the nanoparticle addition to the PAN solution. Moreover, the atomic percentages of Ag, TiO_2_, and ZnO in the obtained filters were much lower than the percentages introduced in the solutions Ag/PAN/DMF, TiO_2_/PAN/DMF, and ZnO/PAN/DMF, respectively. This finding could be explained by the much higher contribution of the substrate (made of PET fibers) to the elemental composition measured by EDX than the thin film of PAN/nanoparticle nanofibers deposited by electrospinning.

Elemental mapping images of Ag_F, TiO_2__F, and ZnO_F filters (Figure S1) showed that nanoparticles were distributed on the whole area of the analyzed samples, indicating their good dispersion in PAN nanofibers. Altogether, these data demonstrate that electrospinning allowed the successful fabrication of Ag/TiO_2_/ZnO nanofibers and their deposition on the PET substrate to produce air filters.

**Figure 2:**
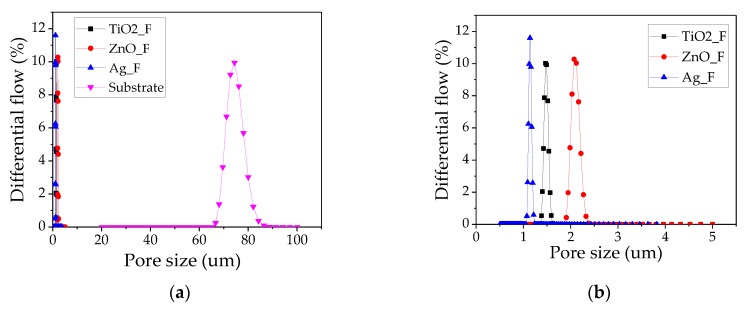
Pore size distribution: (**a**) scale 0–100 μm and (**b**) scale 0–5 μm.

**Figure 3:**
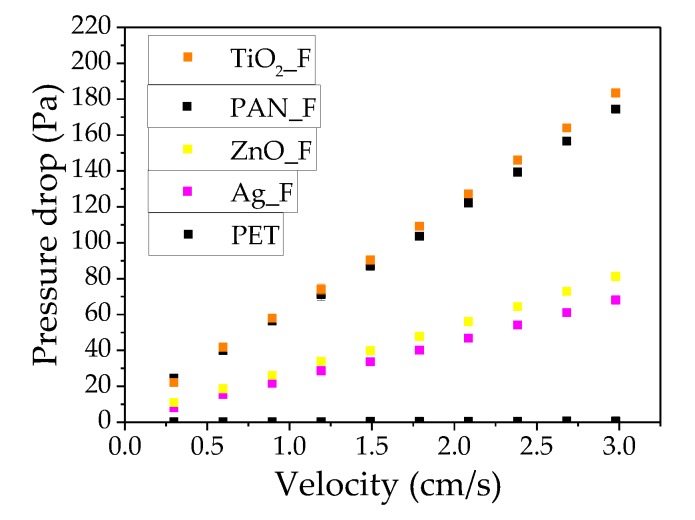
Pressure drop as a function of superficial velocity of electrospun filters.

Pore size and thickness measurements (Figure 2) showed differences in pore size distribution among samples. The narrowest pore size distribution was observed in Ag_F (1.11–1.16 µm; mean value: 1.12 µm) (Table 3) and the largest in ZnO_F (1.99–2.17 µm; mean value: 2.03 µm). Large pore size is correlated with lower pressure drop because the air can easily go through a filter with wide pores. However, in our experimental set up, pressure drop was more influenced by the filter thickness. The lowest pressure drop was observed with ZnO_F and Ag_F filters (Figure 3) that displayed the lowest thickness. As mentioned before, the Ag solution’s viscosity favored the lower fiber deposition compared with the other filters, as previously reported by Li and Wang [50]. TiO_2__F displayed the highest thickness and also the largest pressure drop. This big pressure drop might be explained by the higher amount of nanofibers deposited on the substrate (facilitated by the low TiO_2_ solution viscosity) and nanoparticle agglomeration during electrospinning that hindered the air flow through the filter. Finally, the used substrate (S) did not significantly affect the air flow through the filter because the pressure drop was almost zero and the mean pore size of the substrate was 72.74 µm. Therefore, the substrate did not interfere with the filter performance. On the other hand, the pressure drop increased when PAN_F were deposited on the substrate by electrospinning, probably because increasing the nanofiber layers decreases the void space, hindering air flow through the filter. Conversely, addition of ZnO and Ag nanoparticles to the solution resulted in filters with lower pressure drop due to the higher solution viscosity and the consequent lower nanofiber thickness.

Finally, Ag_F displayed the highest permeability constant (1.83 E^−12^·m^2^) compared with the other filters (Table 4). As permeability is directly proportional to the flow rate, air passed through the Ag_F filter more easily and pressure drop across the filter was reduced (68.13 Pa). This result suggests that adding nanoparticles in the PAN solution beyond a critical value of viscosity hinders the flow of the solution through the needle tip, decreasing the nanofiber deposition and the pressure drop [55,56,57].

### 3.3. Comparison of the Filtration Performance

**Figure 4:**
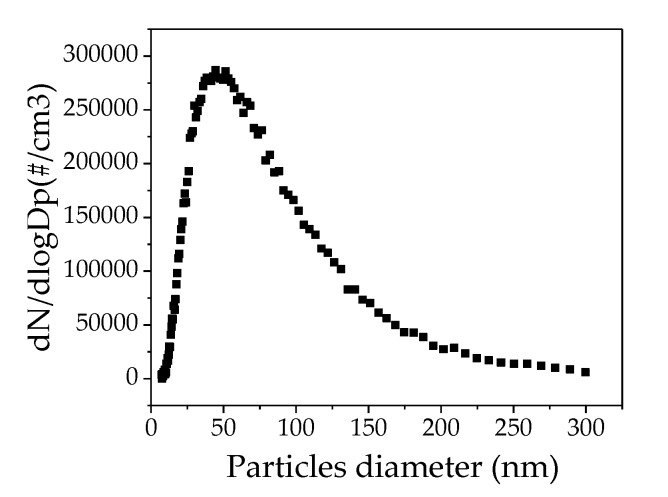
Nanoparticle size distribution using 0.1 g/L NaCl solution.

NaCl nanoparticle with the same size distribution curves (range: 9 to 300 nm) were prepared, to test the different filters, using 0.1 g/L NaCl solution and an atomizer aerosol generator (Figure 4).

**Figure 5:**
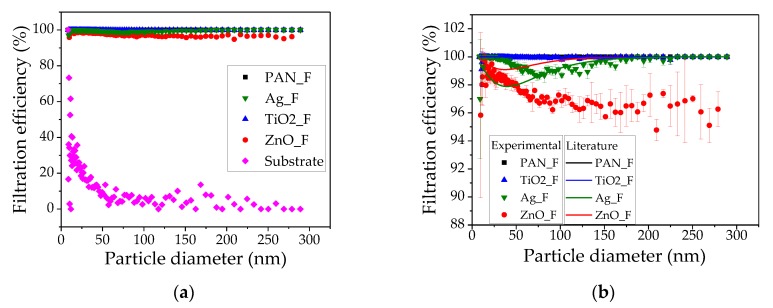
Experimental and theoretical efficiency of the tested filter media: (**a**) scale 0–100% and (**b**) scale 88–102%.

Then, PAN_F, Ag_F, TiO_2__F, and ZnO_F efficiency in removing these nanoparticles (9–300 nm) from the air stream was measured using a differential mobility analyzer and particle counter and compared to the theoretical efficiency (Figure 5). As expected, the substrate efficiency was very low because it was just a support for the nanofibers and did not influence the filtration efficiency (Figure 5a). Therefore, filtration efficiency was analyzed after exclusion of the substrate, which means a smaller scale (Figure 5b). The highest filtration efficiency (≈100%) was observed with TiO_2__F that also displayed the highest air pressure drop (≈183.47 Pa), due to its higher thickness and formation of particle agglomerates on the nanofibers due to TiO_2_ nanoparticle size (21 nm in diameter). This performance can be explained by the large specific surface area and low-ordered crystalline structure of TiO_2_ nanoparticles [58,59]. Zhang et al. [60] demonstrated that TiO_2_ loading on PAN nanofibers enhances particle removal efficiency due to the high surface-charge that improves the particle’s electrostatic attraction. Ag_F also showed high filtration efficiency (>98%), but lower pressure drop (68.13 Pa) compared with TiO_2__F, due to the lower Ag_F thickness, resulting from the lower-fiber deposition on the substrate, and probably the lowest average pore size. Moreover, Ag nanoparticles did not agglomerate on the nanofiber, due to their lower size (<5 nm in diameter). ZnO_F was the least efficient (95% filtration) among the tested filters, but displayed the lowest pressure drop (81.17 Pa). This could be attributed to the larger pore size and lower thickness compared with TiO_2__F. The higher filtration performance of Ag_F despite being less thick than ZnO_F could be explained by the smaller pore size of Ag_F, resulting in better removal of airborne particles compared with ZnO_F. In conclusion, the filter media assessed in this work showed high filtration efficiency, even after nanoparticle loading in the PAN solution.

To understand the different filtration efficiency curve behaviors observed with our filters, the three main capture mechanisms relative to particle size were analyzed: interception, inertial impaction, and diffusion. The filtration curve of our filters is similar to the overall filtration curve for the various filtration mechanisms studied by Hinds (1982) [48], but for ZnO_F and Ag_F. A small deviation is explained by the fiber and particle diameter, thickness, and porosity, which are normally used to calculate the theoretical efficiency. Generally, particles of 0.1–0.4 µm in diameter are considered the most penetrating and can be retained through diffusion and interception filtration. Particles smaller than 0.1 µm in diameter are captured through diffusion. Fibrous filters are generally less effective in removing 0.1 to 0.4 µm particles. Therefore, particles of 9 to 300 nm in diameter are too large to be captured by diffusion and too small to be retained by inertial impaction and interception, and so the filter efficiency decreases within this range [61].

The performance of filter media can be analyzed using the *Q_F_* that was calculated for particles of 100 nm in diameter (Table 5). High *Q_F_* values indicate good filtration efficiency and low pressure drop. In agreement, Ag_F displayed the highest *Q_F_* (pressure drop = 68.13 Pa; filtration efficiency: >98%). TiO_2__F and ZnO_F had similar *Q_F_* values due to the very high pressure drop of TiO_2__F (183.47 Pa), and the low filtration efficiency (>95%) of ZnO_F. The *Q_F_* value of PAN_F (0.05 Pa^−1^) was good, but this filter does not contain bactericidal nanoparticles. The *Q_F_* values of all our filter media, even after addition of TiO_2_, ZnO, and Ag nanoparticles, were comparable to those of previous studies on pristine PAN filters [62,63,64]. However, superficial velocity, particle size, and material composition might vary among studies and thus data should be compared with caution. Wang et al. [52] demonstrated high filtration efficiency (99.97%), low pressure drop (57 Pa) and satisfactory *Q_F_* (0.14 Pa^−1^) using 300–500 nm particles. However, the removal of microparticles from air is usually easier compared with nanoparticles. The nanoparticles of 9 to 300 nm in diameter used in our work are the most difficult to remove and also have been implicated in many diseases [2]. Yet, good *Q_F_* values were obtained (~0.05 Pa^−1^). A relatively small difference was observed because of the slight variation between pressure drop and filtration efficiency for all filters.

On the basis of the European Union Standard for HEPA and ULPA filters—EN 1822 [65], PAN_F filter media could be classified as H13 (HEPA >99.95% collection efficiency), TiO_2__F as E12 (Efficiency Particulate Air Filters–EPA >99.5% collection efficiency), and Ag_F and ZnO_F as E11 (EPA >95% collection efficiency). Following the ISO Cleanroom Standards, PAN_F and TiO_2__F can be classified as ISO Class 3, and Ag_F and ZnO_F could be in ISO Class 4 because the limits of the maximum concentration (1000 particles/m^3^ of air) for particles of 0.1 µm in diameter are exceeded.

### 3.4. Bactericidal Activity

**Figure 6:**
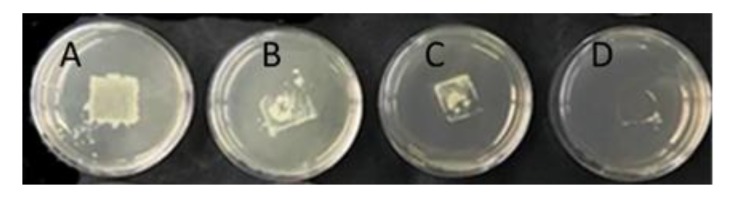
Direct-contact agar tests using 10^8^ CFU/mL *E. coli* suspension (contact for 6 h): (**A**) ZnO_F; (**B**) TiO_2__F; (**C**) PAN_F; and (**D**) Ag_F.

**Figure 7:**
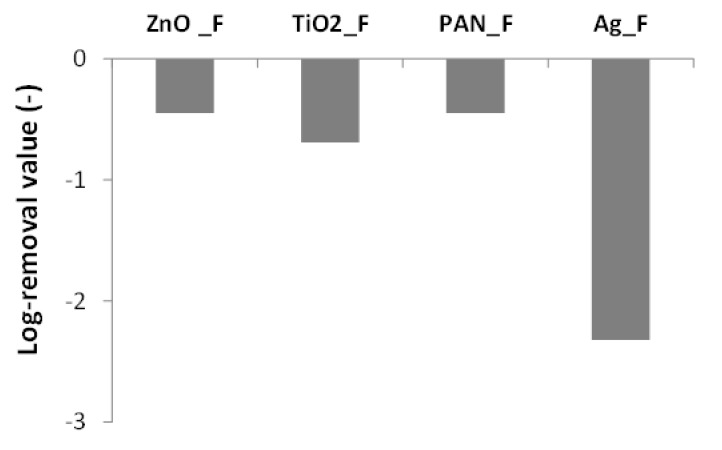
Direct-contact agar tests: log-removal values using a 10^3^ CFU/mL *E. coli* suspension that was in contact with the indicated filters for 6 h. The log-removal was calculated as the logarithm (base 10) ratio of the bacterial quantity Q (CFU) measured after contact with the filter relative to the bacteria quantity *Q_c_* in the positive control.

In the direct-contact agar tests using a 10^8^ CFU/mL *E. coli* suspension, Ag_F clearly showed the highest bactericidal activity because very few bacterial colonies were visible after plate incubation compared with PAN, ZnO_F, and TiO_2__F (Figure 6). Then, the bacterial suspension was decreased to 10^3^ CFU/mL to try to detect the bactericidal activity of the ZnO_F and TiO_2__F materials (Figure 6) and to quantify bacteria removal (Figure 7). For this test, an Ag_F material with lower Ag content (1% instead of 50% *w*/*w*) was used to allow bacteria counting. This experiment confirmed the high bactericidal activity of Ag_F (i.e., removal of all bacteria) (Figure 7) whereas ZnO_F and TiO_2__F did not show any significant antibacterial action. Indeed, the log-removal values were comparable for ZnO_F and TiO_2__F and PAN-F, which unexpectedly displayed a −0.5 log removal. A Student statistical test proved that there is no significant difference among ZnO, TiO_2_, and PAN fibers (*p* > 0.05). Some authors [20,66] showed that PAN nanofibers without antibacterial agents do not have bactericidal activity. Conversely, Bortolassi et al. [39] detected a colony decrease with PAN_F (25 ± 12 CFU, *n* = 3) compared with control (87 ± 10 CFU, *n* = 3), and suggested that bacteria were adsorbed onto PAN material. It can be also hypothesized that when PAN_F (or any other membrane material) is present on the plate surface, a limited O_2_-mass transfer could slightly reduce the growth of the aerobic bacterium tested. Thus, the PAN_F log-removal value was considered as the baseline value for the tests in our study.

The results obtained with Ag_F are consistent with the literature data. It was previously demonstrated that Ag nanoparticles have high antibacterial effectiveness due to their isotropic geometries, such as spherical particles [67,68] that exhibit large surface-to-volume ratio. Indeed, smaller particles can penetrate more easily in bacteria, especially Gram-negative microorganisms [69]. Some studies reported the antibacterial effect of ZnO and TiO_2_ nanoparticles [35,70] without UV illumination [58]. The absence of bactericidal effect of ZnO_F and TiO_2__F in our work, even when using a 10^3^ CFU/mL *E. coli* suspension, could be explained by ZnO agglomeration and the considerable amount of TiO_2_ nanoparticles in the nanofibers (Figure 1). Lv et al. [38] observed that by increasing the concentration of ZnO nanoparticles in PVA and PVA/KGM solutions to more than 1.0 wt%, the solution spinnability was drastically reduced. Moreover, ZnO particles clustered together and adhered to the fibrous membranes in a random manner. They then showed that the antibacterial activity of a membrane with random ZnO clusters was lower than that of a membrane with uniformly dispersed ZnO.

Our results indicate that loading silver nanoparticles in PAN nanofibers using electrospinning is an efficient method to develop air filters for airborne nanoparticle removal with bactericidal activity. They indeed highlight the efficient antibacterial activity, lowest pressure drop (68.13 Pa), high filtration efficiency (>98%), highest *Q_F_* (0.06 Pa^−1^), and highest permeability of Ag_F nanofibers, which also displayed the lowest nanofiber deposition during electrospinning.

## 4. Conclusions

In this study, TiO_2_/PAN, ZnO/PAN, and Ag/PAN nanofibers electrospun using the same weight ratio and the same experimental conditions were evaluated. The PAN solution viscosity and conductivity was modified upon addition of the nanoparticles and this affected the nanofiber formation, although they were produced using the same electrospinning parameters. Specifically, viscosity and conductivity were comparable in TiO_2_/PAN/DMF and ZnO/PAN/DMF solutions, whereas they were much higher for the Ag/PAN/DMF solution (933 cP and 2.11 mS/cm, respectively). SEM images confirmed the formation of nanofibers and the homogeneous Ag dispersion on the fibers. On the other hand, TiO_2_ and ZnO nanoparticles formed agglomerates on the fibers. Characterization of the filter thickness, pore size, pressure drop, and permeability indicated an overall low pressure drop (from 68.13 to 183.47 Pa). The highest filtration efficiency (≈100%) was obtained with the TiO_2__F filter, but it also displayed the biggest pressure drop (≈183.47 Pa), probably because of the great number of nanofibers deposited on the substrate. Ag_F showed high filtration efficiency (>98%), low pressure drop (68.13 Pa), high *Q_F_* (0.06 Pa^−1^), and very good antibacterial activity against a 10^8^ CFU/mL *E. coli* suspension. ZnO_F displayed the lowest filtration efficiency, which was nevertheless >97%. Therefore, our work shows that filter media maintain high filtration efficiency even after the addition of nanoparticles in the PAN solution. It also suggests that Ag/PAN nanofiber media could be used for many air filtration applications (e.g., masks, cleanrooms, and indoor air purification), thanks also to their bactericidal activity.

## Figures and Tables

**Table 1:** Conductivity and viscosity measured at 25 °C.

Solutions	Conductivity (mS/cm)	Viscosity (cP)
PAN/DMF	0.09 ± 0.01	471 ± 0
Ag/PAN/DMF	2.11 ± 0.05	933 ± 1
TiO_2_/PAN/DMF	0.09 ± 0.01	452 ± 1
ZnO/PAN/DMF	0.08 ± 0.01	567 ± 1

**Table 2:** Characterization of the electrospun fibrous filters and unwoven substrate.

Samples	PAN (g)	Nanoparticles (g)	Mean Fiber Diameter (nm)	Thickness (mm)	Basis Weight (g/m^2^)
PAN_F	0.95	–	301 ± 7	0.20 ± 0.01	75 ± 3
Ag_F	0.95	0.95	292 ± 6	0.17 ± 0.01	62 ± 4
TiO_2__F	0.95	0.95	242 ± 5	0.19 ± 0.01	79 ± 3
ZnO_F	0.95	0.95	289 ± 5	0.18 ± 0.01	80 ± 3
S	0	0	27 ± 0	0.16 ± 0.01	61 ± 1

**Table 3:** Pressure drop and pore size of the electrospun filters.

Samples	ΔP at 0.03 m/s (Pa)	Pore Size (µm)		
		Minimum	Mean	Maximum
PAN_F	174.50 ± 0.25	1.97 ± 0.10	2.35 ± 0.10	2.93 ± 0.10
Ag_F	68.13 ± 0.18	1.11 ± 0.10	1.12 ± 0.10	1.16 ± 0.10
TiO_2__F	183.47 ± 0.03	1.42 ± 0.10	1.45 ± 0.10	1.51 ± 0.10
ZnO_F	81.17 ± 0.07	1.99 ± 0.10	2.03 ± 0.10	2.17 ± 0.10
Substrate	0.60 ± 0.00	69.59 ± 0.10	72.74 ± 0.10	80.04 ± 0.10

**Table 4:** Permeability constant of the electrospun filters.

Samples	K_1_ (m^2^)
PAN_F	6.11 × 10^−13^
Ag_F	1.83 × 10^−12^
TiO_2__F	6.11 × 10^−13^
ZnO_F	9.17 × 10^−13^
Substrate	1.46 × 10^−10^

**Table 5:** Quality factor of the different filters.

Samples	Quality Factor (Pa^−1^)
PAN_F	0.05
Ag_F	0.06
TiO_2__F	0.04
ZnO_F	0.04

## Supplementary Materials

The following are available online at https://www.mdpi.com/2079-4991/9/12/1740/s1, Figure S1: Elemental mapping images of Ag/TiO_2_/ZnO-PAN nanofibers (**a**) Ag_F; (**b**) TiO_2__F; (**c**) and ZnO_F. Table S1: EDX data showing the composition of PAN_F, Ag_F, TiO_2__F, and ZnO_F filters and the substrate.
