# Supplementary material for: Composites Based on Nanoparticle and Pan Electrospun Nanofiber Membranes for Air Filtration and Bacterial Removal
Source: Nanomaterials (Basel). 2019 Dec 6;9(12):1740. doi: 10.3390/nano9121740 (PMC6956291; doi:10.3390/nano9121740)
Supplement: Supplementary file 1 [file nanomaterials-09-01740-s001.pdf]

*Suporting information*

# Composites Based on Nanoparticle and Pan Electrospun Nanofiber Membranes for Air Filtration and Bacterial Removal

**Ana Cláudia Canalli Bortolassi [1], Vádila Giovana Guerra [1], Mônica Lopes Aguiar [1], Laurence Soussan [2], David Cornu [2], Philippe Miele [2] and Mikhael Bechelany [2,*]**

[1] Departamento de Engenharia Química, Universidade Federal de São Carlos–UFSCar, Rodovia Washington Luiz, km 235–SP 310, 13565-905 São Carlos, Brazil; acanallibortola@deakin.edu.au (A.C.C.B.); vadila@ufscar.br (V.G.G.); mlaguiar@ufscar.br (M.L.A.)

[2] Institut Européen des Membranes, IEM – UMR 5635, ENSCM, CNRS, Univ Montpellier, Montpellier, France; laurence.soussan@univ-montp2.fr (L.S.); david.cornu@enscm.fr (D.C.); philippe.miele@umontpellier.fr (P.M.)

* Correspondence: mikhael.bechelany@umontpellier.fr; Tel.: + 33-0467-149-167

The energy dispersive X-ray spectrum (EDX) analysis reveals the atomic percentages of Ag/Ti/Zn of the fibers developed in this study and are presented in

Table **S1**. The results are in good agreement with the addition of the nanoparticles into the PAN solution. Figure S1 shows elemental mapping images of Ag_F, $TiO_2$_F, and ZnO_F filters. It is possible to notice that these particles are well distributed over the entire area of the sample confirming the good dispersion of bactericidal nanoparticles in PAN nanofibers. Based on these data, Ag/$TiO_2$/ZnO nanofibers were successfully fabricated and deposited in PET substrate using electrospinning method to produce air filters. We can notice in

Table **S1** that the atomic percentages of silver in the obtained filters are much lower that the percentages introduced in the experimental section. This observation could be related to the contribution of the substrate (made of Polyethylene terephthalate (PET) fibers) to the elemental composition measured by EDX due to the thin film of NPs/PAN nanofibers deposited by electrospinning (on the substrate).

**Table S1.** EDX data showing the composition of PAN_F, Ag_F, $TiO_2$_F, ZnO_F filters and the substrate.

| **Samples** | **Atomic Percentage** | | | | | |
|---|---|---|---|---|---|---|
| | **Ag** | **Ti** | **Zn** | **C** | **N** | **O** |
| PAN_F | 0±0 | 0±0 | 0±0 | 74±1 | 23±1 | 2±1 |
| Ag_F | 8±1 | 0±0 | 0±0 | 531 | 30±1 | 9±1 |
| $TiO_2$_F | 0±0 | 7±1 | 0±0 | 65±1 | 13±1 | 13±1 |
| ZnO_F | 0±0 | 0±0 | 6±1 | 70±1 | 18±1 | 6±1 |
| Substrate | 0±0 | 0±0 | 0±0 | 63±4 | 2±1 | 17±5 |

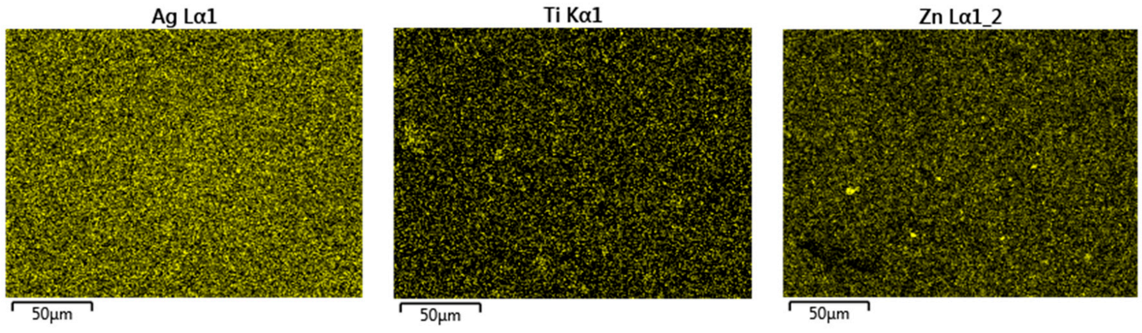


(a) (b) (c)

Figure S1. Elemental mapping images of Ag/$TiO_2$/ZnO-PAN nanofibers (**a**) Ag_F (**b**) $TiO_2$_F (**c**) ZnO_F.
